# Metallic versus biodegradable suture anchors for rotator cuff repair: a case control study

**DOI:** 10.1186/s12891-019-2834-3

**Published:** 2019-10-25

**Authors:** Umile Giuseppe Longo, Stefano Petrillo, Mattia Loppini, Vincenzo Candela, Giacomo Rizzello, Nicola Maffulli, Vincenzo Denaro

**Affiliations:** 1 Department of Orthopaedic and Trauma Surgery, Campus Bio-Medico University, Via Alvaro del Portillo, 200, 00128 Trigoria, Rome, Italy; 2 Centro Integrato di Ricerca (CIR) Campus Bio-Medico University, Via Alvaro del Portillo, 21, 00128 Rome, Italy; 3 Department of Biomedical Sciences, Humanitas University, Via Rita Levi Montalcini 4, 20090 Pieve Emanuele, Milan, Italy; 4 Department of Orthopaedic and Trauma Surgery, Humanitas Clinical and Research Center, Via Alessandro Manzoni 56, 20089 Rozzano, Milan, Italy; 5 Department of Musculoskeletal Disorders, Faculty of Medicine and Surgery, University of Salerno, Salerno, Italy; 6 Centre for Sports and Exercise Medicine, Barts and The London School of Medicine and Dentistry, Mile End Hospital, 275 Bancroft Road, London, E1 4DG England

**Keywords:** Suture anchors, Rotator cuff, Arthroscopy, Metal, Biodegradable

## Abstract

**Backgrounds:**

Repair of full-thickness rotator cuff (RC) tears is routinely performed using suture anchors, which produce secure and effective soft tissue fixation to bone. The aim of this prospective study is to compare the long-term outcomes of single row arthroscopic rotator cuff repair (RCR) performed using metal or biodegradable suture anchors. The null hypothesis is that there is no difference in shoulder function using metal or biodegradable suture anchors as evaluated by UCLA shoulder score, Wolfgang criteria, and Oxford shoulder score.

**Methods:**

Arthroscopic RCR was performed in 110 patients included in this case control study. They were divided into 2 groups of 51 and 59 patients respectively. Metal suture anchors were used in group 1, and biodegradable suture anchors in group 2. Results were obtained at a mean follow up of 4.05 + 2 years. Clinical outcomes and functional outcomes were evaluated.

**Results:**

The mean modified UCLA shoulder score was 26.9 + 7.1 in group 1, and 27.7 + 6.5 in group 2 (*P* = 0.5); the mean Wolfgang score was 13.3 + 3.3 in group 1, and 14 + 2.6 in group 2 (*P* = 0.3); the mean OSS was 23.7 + 11.4 in group 1, and 20.7 + 9.2 points in group 2 (*P* = 0.1). The mean active anterior elevation was 163.5° + 28.2° in group 1 and 163.6° + 26.9 in group 2 (*P* = 0.9); the mean active external rotation was 46° + 19.7° in group 1 and 44.6° + 16.3° in group 2 (*P* = 0.7). The mean strength in anterior elevation was 4.8.02 + 23.52 N in group 1, and 43.12 + 17.64 N in group 2 (*P* = 0.2); the mean strength in external rotation was 48.02 + 22.54 N in group 1 and 46.06 + 17.64 N in group 2 (*P* = 0.6); the mean strength in internal rotation was 67.62 + 29.4 N in group 1, and 68.6 + 25.48 N in group 2 (*P* = 0.9).

**Conclusions:**

There are no statistically significant differences at a mean follow-up of 4.05 + 2 years in clinical and functional outcomes of single row arthroscopic RCR using metallic or biodegradable suture anchors for RC < 5 cm.

## Background

Repair of full-thickness rotator cuff (RC) tears is routinely performed using suture anchors, which produce secure and effective soft tissue to bone repair [1–3]. Outcome of RC surgery is unpredictable, because the biological process that leads the tendon to reattach to the bone have not been clearly identified [4–10]. Metal suture anchors ensure a safe and long-term fixation while biodegradable suture anchors provide fixation for a short period, during which the tissue heal [11].

Metal suture anchors provide a good long term fixation but are often associated with well documented complications such as migration, chondral damage, imprisonment of the anchor within the joint, major technical difficulty with revision surgery and problems with magnetic resonance (MRI) imaging [12–14].

The use of biodegradable implants in arthroscopic rotator cuff repair (RCR) procedures is relatively recent. In fact, biodegradable anchors avoid the potential risk of metal anchors of bone resorption and implant dislodgement [15, 16]. Other advantages of biodegradable over metal implants include less postoperative MRI artifacts and easier revision surgery [17, 18]. Nevertheless, three main disadvantages are associated with the use of biodegradable suture anchors: higher costs, undesired biological response [19] and shorter fixation time. Moreover, biomechanical studies demonstrated that metal anchors present a better fixation strength when compared with biodegradable anchors [20]. Several clinical studies reported excellent results with the use of biodegradable implants, fully superimposable to those obtained with non-absorbable devices [11, 21–23]. However, only one short term follow up randomized control trial [24] compared the clinical and functional outcome of arthroscopic RCR performed with metal or biodegradable suture anchors. The aim of the study is to compare the long-term clinical outcomes of arthroscopic RCR performed with metal or biodegradable suture anchors.

The null hypothesis is that there is no difference in shoulder function using metal or biodegradable suture anchors as evaluated by UCLA shoulder score, Wolfgang criteria, and Oxford shoulder score.

## Methods

Our institutional ethics review board approved the study.

### Type of study

Case control study

### Eligibility criteria

Patients were included in the study if they underwent arthroscopic RCR and the following conditions were present at the time of surgery: RC tear, absence of shoulder instability, absence of shoulder’s fractures, MRI evidence of full-thickness RC tear, duration of symptoms of at least 3 months, inadequate response to non-operative management (including non-steroidal anti-inflammatory drugs, physiotherapy, rest, and one local corticosteroid injection), a repairable RC tear found at the time of surgery. Patients with pathology of the tendon of the long head of the biceps were also included in the study.

Exclusion criteria were: inflammatory joint disease, prior surgery on the affected shoulder, labral pathology, degenerative arthritis of the glenohumeral joint, symptomatic arthritis of the acromioclavicular joint, RC arthropathy, inability to complete questionnaires.

In 51 patients, RCR was performed using metallic suture anchors (Corkscrew, Arthrex, Naples, FL) (Group 1), while in 59 patients RCR was performed using biodegradable suture anchors (Biocorkscrew, Arthrex, Naples, FL) (Group 2). Of the 110 patients enrolled in the present investigation, results at an average of 4.05 + 2 year were available for 108 patients. Two patients, both in group 1, were excluded from the study because of cognitive disorders. All tears were < 5 cm in size in both group.

### Evaluation

The mean follow up period were 4.05 + 2 year (range 1 to 10 years) from the surgery. Age; sex; arm dominance; history of trauma; location of RC tear; dimension of the RC tear; biceps tendon rupture or tendinopathy; type of treatment of biceps tendon; acromioplasty; number of anchors used; post-operative range of motion (ROM); post-operative modified University of California, Los Angeles (UCLA) [25] shoulder rating scale; post-operative Wolfgang criteria shoulder score; post-operative Oxford shoulder score (OSS) [26, 27]; post-operative strength of anterior elevation, external and internal rotation were evaluated.

### Clinical assessment

A modified UCLA [25] shoulder rating scale was used to evaluate strength (5 points), shoulder pain (10 points), function (10 points), active forward flexion (5 points) and patient satisfaction (5 points). The maximum score obtainable is 35, and the results were classified as excellent (34–35 points), good (28–33), fair (21–27), or poor (0–20).

The Wolfgang criteria were used to assess post-operative shoulder pain (4 points), active abduction (4 points), strength (4 points) and patient satisfaction (1 point or minus 1 point). The maximum score obtainable is 17, and the results were classified as excellent (14–17 points), good (11–13 points), fair (8–10 points) or poor (0–7 points).

Postoperatively, all patients completed the Italian version of the (OSS) [26], a questionnaire that evaluates shoulder function, pain and strength in relationship with daily life activities. The minimum score is 12 points and the maximum score is 60 points. The higher is the score, the worse the condition of the shoulder.

### Range of motion and strength

Clinical and functional evaluations were performed by two blinded examiners. Patients were positioned supine with the shoulder at 90° of abduction in the scapular plane. Supine passive and active forward elevation (sagittal plane), internal and external rotation ROM (90° abduction) were evaluated with a standard universal goniometer [28].

A dynamometer (mod.CH 15 K20-KERN Balingen - Germany) was used to measure the strength of anterior elevation, internal and external rotation of the shoulder, and the results obtained were expressed in Newton (N). Both examiners performed three measurements for each ROM and strength measurement investigated. The average value for each variable was used for statistical purposes.

### Sample size and demographic details

Patients were divided into two groups: metallic suture anchors (Group 1), or biodegradable suture anchors (Group 2). Demographic and surgical details of the patients enrolled in the study are shown in Table 1.

**Table 1:** Demographic and surgical details

VARIABLE	GROUP 1	GROUP 2	*P*
AGE (mean + sd)	56.5 + 10	58.1 + 9	0.5
SEX			1
Male	26	27	
Female	23	32	
ARM DOMINANCE			0.2
Yes	40	42	
No	9	17	
TRAUMA			0.3
Yes	12	10	
No	37	49	
LOCATION			1
Supraspinatus	25	30	
Supraspinatus+ Infraspinatus	24	29	
DIMENSION			0.1
< 1 cm or 1–3 cm	25	41	
3–5 cm	24	18	
LHB patology			0.2
Tear	18	24	
Tendinophaty	10	14	
Absent	21	11	
LHB Treatment			0.3
No	17	19	
Tenotomy	19	25	
Tenodesis	13	5	
ACROMIOPLASTY			
Performed	22	29	
Not performed	27	30	
NUMBER OF ANCHORS (mean + sd)	1.71+ 0.72	1.76 + 0.67	0.5

*Sd* Standard Deviation, *LHB* Long head biceps

### Arthroscopic technique

Arthroscopic RCRs were performed by two orthopaedic surgeons, expert in the use of both metallic and biodegradable implants.

Patients underwent brachial plexus block (associated, in 21 patients, with general anaesthesia). RCRs were performed with patients in a lateral decubitus position and the affected arm at approximately 45° of abduction and 20° of forward flexion. Distraction of the shoulder joint was accomplished with 4.5 to 6.5 kg of traction. A diagnostic arthroscopy was made. Bleeding was controlled using radiofrequency and adrenalin mixed to the irrigation fluid. A subacromial decompression was performed in the presence of a type III Acromion.

Footprint of the greater tuberosity was abraded. RCR was performed placing one row of suture anchors double loaded with N° 2 Fiberwire (Corkscrew, Arthrex, Naples, FL) (Group 1) or (Biocorkscrew, Arthrex, Naples, FL) (Group 2) just in the lateral aspect of the footprint. The number of suture anchors varied with the size of the tear. We used 2 or 3 suture anchors with a single row technique in patients with a tear larger than 3 cm and 1 suture anchor in patients with a tear < of 3 cm.

### Post-operative management

The two groups had the same post-operative management. Patients used a sling for the affected arm with an abduction pillow for 6 weeks. Movements allowed were: active elbow flexion; active elbow extension; passive external rotation. Terminal extension and overhead stretching were restricted until 6 weeks post-operatively and manual work and overhead activities were restricted until 12 months. Strengthening and rehabilitation of the rotator cuff, deltoid and scapular muscles were initiated at 10 or 12 weeks after surgery.

### Statistics

Statistical analyses were conducted considering the following outcome scores: total modified UCLA shoulder score, total Wolfgang criteria shoulder score and total OSS. We considered also active and passive ROM and strength of anterior elevation, internal rotation and external rotation. The independent variables analyzed were: age; sex; arm dominance; history of trauma; location and dimension of the rotator cuff tear; biceps tendon rupture or tendinopathy; type of treatment of biceps tendon; acromioplasty; number of anchors used. Comparison between the 2 groups for each independent variable was carried out with the Student *t* test for continuous variables and the *×* 2 test for categorical variables. The outcome variables considered (shoulder outcome scores, active and passive ROM, muscle strength) were compared using the Student *t* test. A one-way ANCOVA was performed to compare the effectiveness of the type of suture anchors on outcome scores, ROM and strength whilst controlling for the length of follow-up. The effect size was evaluated according Cohen’s guidelines: 0.2 - small effect, 0.5 - moderate effect, 0.8 - large effect. Significance was set at *P* < 0.05.

### Sample size calculation

In the present study, we enrolled a series of 110 consecutive patients who underwent RC repair at our institution without an a priory power analysis. However, we undertook a post hoc power analysis using UCLA score. According to previous study, the UCLA shoulder score minimal clinically important difference is 2.96 points in the 35-point scale [29]. We estimated that our study had 90% of power with an alfa error probability of 0.05, an effect size of 0.65 and a minimum of 50 patients for each group.

## Results

From 197 potentially eligible patients, 180 decided to be examined for eligibility and 110 met the eligible criteria to be enrolled in this study. The mean follow-up was 4.05 + 2 years (range 1–10). As 2 patients from group 1 were excluded, the final evaluation includes 108 patients (53 men and 55 women; mean age 57.3 + 9.5 years, range 29–76). No patient experienced infection, neurological or vascular complications after surgery. The comparison between groups did not show significant differences between them for each independent variable considered **(**Table 2**).**

**Table 2:** Outcome scores

Outcome scores	MEAN + STANDARD DEVIATION		*P*
	GROUP 1	GROUP 2	
UCLA	26.9 + 7.1	27.7 + 6.5	0.5
WOLFGANG	13.3 + 3.3	14 + 2.6	0.3
OSS	23.7+ 11.4	20.7 + 9.2	0.1

*OSS* Oxford shoulder score

According to the results of our study, at a mean follow-up of 4.05 + 2 years, clinical and functional differences between arthroscopic RCR performed with metal or biodegradable suture anchors are not significant (*P* < 0.5).

Sixty-four subjects underwent RC repair using 1 suture anchor. 44 subjects underwent RC repair using 2 or more suture anchors. Overall, the mean modified UCLA shoulder score was 24.7 + 4.9 points in subjects who underwent RC repair using 1 suture anchor, and 25.5 + 4.3 in who underwent RC repair using 2 or more suture anchors (*P* < 0.5); the mean Wolfgang criteria scores was 12.2 + 2.2 points in group who underwent RC repair using 1 suture anchor, and 13 + 1.5 in group who underwent RC repair using 2 or more suture anchors (*P* < 0.5); the mean Oxford shoulder scores were 20.4 + 8.1 points in group who underwent RC repair using 1 suture anchor, and 17.4 + 5.9 in group who underwent RC repair using 2 or more suture anchors (*P* < 0.5).

The mean passive anterior elevation was 166.1° + 20.3 in group who underwent RC repair using 1 suture anchor, and 179.6° + 13.6 in group who underwent RC repair using 2 or more suture anchors (*P* < 0.5); the mean passive external rotation was 71.7° + 21° in group who underwent RC repair using 1 suture anchor, and 72.2° + 18° in group who underwent RC repair using 2 or more suture anchors (*P* < 0.5); the mean passive internal rotation was 82.1° + 12.9° in group who underwent RC repair using 1 suture anchor, and 81.3° + 13.7° in group who underwent RC repair using 2 or more suture anchors (*P* < 0.5).The mean active anterior elevation was 162.4° + 27.1° in group who underwent RC repair using 1 suture anchor, and 164.5° + 25.8 in group who underwent RC repair using 2 or more suture anchors (*P* < 0.5); the mean active external rotation was 44° + 17.7° in group who underwent RC repair using 1 suture anchor, and 42.6° + 14.3° in group who underwent RC repair using 2 or more suture anchors (*P* < 0.5).

The mean strength in anterior elevation was 44.02 + 19.52 N in group who underwent RC repair using 1 suture anchor, and 39.12 + 13.64 N in group who underwent RC repair using 2 or more suture anchors (*P* < 0.5); the mean strength in external rotation was 43.02 + 20.54 N in group who underwent RC repair using 1 suture anchor and 41.06 + 15.64 N in group who underwent RC repair using 2 or more suture anchors (*P* < 0.5); the mean strength in internal rotation was 66.61 + 28.4 N in group who underwent RC repair using 1 suture anchor, and 67.5 + 24.48 N in group who underwent RC repair using 2 or more suture anchors (*P* < 0.5).

No statistical differences were found in terms of functional outcome using 1 or 2 or more suture anchors (*P* < 0.5);

### Group 1 (metallic suture anchors)

49 subjects underwent RC repair with metallic suture anchors. The average of postoperative modified UCLA shoulder score was 26.9 + 7.1 points (range 6–35). According to the modified UCLA shoulder rating system, in 7 patients (14%) the results were considered excellent, in 22 patients (46%) good, in 10 patients (20%) fair, and in the remaining 10 patients (20%) poor.

The average of postoperative Wolfgang criteria shoulder score was 13.3 + 3.3 points (range 2–17). According to Wolfgang criteria shoulder score, in 29 patients (59%) the results were considered excellent, in 11 patients (22%) were good, in 6 (12%) patients were fair, and in the remaining 3 patients (6%) poor.

The average of postoperative OSS was 23.7 + 11.4 points (range: 12–51)**.**

The mean postoperative passive and active forward flexion, external and internal rotation ROM is reported in Table 3**.** Table 3 also lists the mean strength value in anterior elevation, external and in internal rotation ROM.

**Table 3:** Group 1 functional results

Group 1	PASSIVE ROM			ACTIVE ROM		STRENGHT (N)		
Metallic	A.E.	E.R.	I.R.	A.E.	E.R.	A.E.	E.R.	I.R.
MEAN	169.4	73.9	84.3	163.5	46	48.02	48.02	67.62
ST.D	23.6	23	15.1	28.2	19.7	23.52	22.54	29.4
MAX	180	90	90	180	90	107.8	107.8	107.8
MIN	90	10	30	90	0	19.6	14.7	9.8

*ROM* range of motion, *AE* anterior elevation, *ER* external rotation, *IR* internal rotation

Four of the patients in this group were involved in recreational volleyball, and 3 in recreational soccer: all returned to their pre-injury levels of sports.

### Group 2 (biodegradable suture anchors)

The average postoperative modified UCLA shoulder score was 27.7 + 6.5 points (range 6–35). \**.** According to the modified UCLA shoulder rating scale, 12 patients (20%) were excellent, 22 patients (37%) were good, 17 (29%) were fair, and the remaining 8 patients (14%) were poor.

The average of postoperative Wolfgang criteria shoulder score was 14 + 2.6 points (range 4–17). According to the Wolfgang criteria shoulder score, 35 patients (60%) were considered excellent, 20 patients (34%) good, 2 (3%) fair, and the remaining 2 patients (3%) poor.

The average of postoperative OSS was 20.7 + 9.2 points (range: 12–46).

The mean postoperative passive and active forward flexion, external and internal rotation ROM is reported in Table 4**.** Table 4 also lists the mean strength value in anterior elevation, external and in internal rotation ROM.

**Table 4:** Group 2 functional results

Group 2	PASSIVE ROM			ACTIVE ROM		STRENGHT (N)		
Biodegradable	A.E.	E.R.	I.R.	A.E.	E.R.	A.E.	E.R.	I.R.
MEAN	173.1	74.4	83.5	163.6	44.6	43.12	46.06	68.6
ST.D	19.9	20	15.9	26.9	16.3	21.56	17.64	25.48
MAX	180	90	90	180	90	107.8	102.9	107.8
MIN	100	20	30	90	15	9.8	16.66	3

*ROM* range of motion, *AE* anterior elevation, *ER* external rotation, *IR* internal rotation

Two of the patients in this group were involved in recreational tennis, and both returned to their pre-injury levels of sports.

### Group 1 vs group 2

The comparison between the two groups did not show any statistically significant differences for any of the outcome variable considered.

Overall, the mean modified UCLA shoulder score was 26.9 + 7.1 points in group 1, and 27.7 + 6.5 in group 2 (*P* = 0.5); the mean Wolfgang criteria scores was 13.3 + 3.3 points in group 1, and 14 + 2.6 in group 2 (*P* = 0.3); the mean Oxford shoulder scores were 23.7 + 11.4 points in group 1, and 20.7 + 9.2 in group 2 (*P* = 0.1).

The mean passive anterior elevation was 169.4° + 23.6 in group 1, and 173.1° + 16.9 in group 2 (*P* = 0.3); the mean passive external rotation was 73.9° + 23° in group 1, and 74.4° + 20° in group 2 (*P* = 0.9); the mean passive internal rotation was 84.3° + 15.1° in group 1, and 83.5° + 15.9° in group 2 (*P* = 0.8). The mean active anterior elevation was 163.5° + 28.2° in group 1, and 163.6° + 26.9 in group 2 (*P* = 0.9); the mean active external rotation was 46° + 19.7° in group 1, and 44.6° + 16.3° in group 2 (*P* = 0.7).

The mean strength in anterior elevation was 48.02 + 23.52 N in group 1, and 43.12 + 17.64 N in group 2 (*P* = 0.2); the mean strength in external rotation was 48.02 + 22.54 N in group 1 and 46.06 + 17.64 N in group 2 (*P* = 0.6); the mean strength in internal rotation was 67.62 + 29.4 N in group 1, and 68.6 + 25.48 N in group 2 (*P* = 0.9).

## Discussion

This study compared the long-term clinical and functional outcomes of patients who underwent arthroscopic RCR using metal or biodegradable suture anchors.

The findings of our study are similar to those reported in a randomized controlled trial [24] comparing the outcomes at a short-term follow up of RCR performed using biodegradable or metallic suture anchors.

The first types of suture anchors used for RCR were metallic. However, they may be associated with well documented complications such as migration, incarceration of the metal implant within the joint, chondral damage, loosening and technical difficulty with revision surgery [12–14]. Mobilization of metal implants can be identified at radiography [30]. Moreover, metal implants can produce artefacts in MRI studies.

Biodegradable suture anchors have been used since the 1990s. Biodegradable devices provide the necessary initial strength, and their mechanical characteristics during degradation decline slowly. This allow appropriate healing [31]. Moreover, the pullout strength of biodegradable suture anchors is comparable to that of metal anchors [32].

However, biodegradable suture anchors present disadvantages when compared with metallic implants, including higher costs, limited fixation time and severe major complications. One complication described is foreign-body reaction, which ranges from mild fluid accumulation to sterile discharging sinuses to irreversible tissue damage. In these cases, histopathology has consistently shown sterile, nonspecific inflammatory response [19]. Anchor dislodgement was reported in 13 of 30 patients with pain after RCR with biodegradable anchors [30], while in other studies disintegration of the implant was reported [16], and also humeral head osteolysis [15]. However, complications associated with biodegradable suture anchors are relatively uncommon, and with only few cases out of hundreds of thousands of implanted anchors.

From a clinical point of view, there is a lack of information about the differences between metallic and biodegradable suture anchors in RCR. Arthroscopic repair is well established for the management of RC tears, and many different techniques of repair have been described. Nevertheless, only few studies focused on the comparison between different implant materials. This information can be useful for the surgeons such as the institutions.

Although the range of follow-up is quite large, no significant statistical difference between long and short term patients was found. In our analysis, we considered three different shoulder scores (UCLA, Wolfgang and OSS), such as active and passive ROM and muscles strength. The UCLA shoulder score, Wolfgang criteria and OSS were higher in group 2 while active ROM was better in group 1. Moreover, the strength of anterior elevation and external rotation was better in group 1 while the strength of internal rotation was better in group 2. However, none of these differences were statistically significant.

Major strengths of the present study are that two fully trained surgeons performed all the operations using a well-established technique and the same type of metal or biodegradable suture anchors. In addition, the follow up evaluations were performed by two independent blinded assessors following standard measurements guidelines [33]. Our follow up, at an average of 4.05 years, is long enough to consider that, by then, the results of surgery had stabilised, the repaired tissue healed and the function and muscle strength were recovered.

Limitations of the study are that we did not perform post-operative imaging evaluation in our patients, and that we have very large range of follow-up. However, our previous study in this field showed that, although there is a definite rate of post-repair rotator cuff failure, this is often asymptomatic, and therefore post-operative imaging in and by itself should not be considered a measure of failure [34–37]. We are mounting further studies in which we plan to include imaging as an outcome measure. Bioabsorbable anchors may produce inflammatory reactions, with a reactive synovitis associated with pain and stiffness that does not respond to the use of anti-inflammatory drugs [38]. It would be interesting to know, in future studies, whether these imaging features are correlated with clinical outcome.

Rotator cuff surgery aims to provide tendon fixation secure enough to hold the repaired tendon in place until biological healing occurs [34–37]. Several factors may be implicated in failure of rotator cuff repairs, including suture or knot failure, inadequate tendon to bone fixation, and lack of tendon to bone healing. However, biodegradable implants present important disadvantages.

Based on the results of the present investigation, we routinely use metallic suture anchors in patients undergoing arthroscopic RCR, to reduce costs of the operations and risk of severe complications. In our hands, biodegradable and metallic suture anchors provide similar clinical and functional outcomes for RCR. Obviously, we have to specify that our results refer to a single type of biodegradable or metal suture anchor. A biomechanical study [39] demonstrated that subtle design differences can affect the mechanical behavior of biodegradable suture anchors, and therefore our results cannot be translated to all implants available.

Additional biomechanical studies and appropriately powered randomized controlled trials with long term follow up are needed to better understand the real advantage of biodegradable over metal implants in arthroscopic RCR procedures.

According to the ANCOVA analysis, there was a significant difference in mean values of active and passive ROM and strength of anterior elevation, internal rotation and external rotation (Table 5). On the other hand, there was no significant difference in mean values of UCLA, Wolfgang and OSS scores (Table 5).

**Table 5:** ANCOVA analysis for active and passive ROM and strength of anterior elevation, internal rotation and external rotation between the group 1 and 2

Dependent variable	F (Test Statistic)	*P* value	Partial Eta Squared
OSS	1985	0.162	0.131
UCLA	0.365	0.547	0.003
WS	1166	0.283	0.011
Passive ROM AE	6225	0.014	0.056
Passive ROM ER	1,422,404	< 0.0001	0.931
Passive ROM IR	1,889,092	< 0.0001	0.947
Active ROM AE	13,645	< 0.0001	0.115
Active ROM ER	2,798,922	< 0.0001	0.964
Strength AE	310,575,137	< 0.0001	1.0
Strength ER	388,582,105	< 0.0001	1.0
Strength IR	214,173,738	< 0.0001	1.0

## Conclusion

There are no statistically significant differences at a mean follow-up of 4.05 + 2 years in clinical and functional outcomes of single row arthroscopic RCR using metallic or biodegradable suture anchors for RC < 5 cm. Moreover, passive and active ROM of the shoulder and RC muscles strength show no statistically significant differences between metal or biodegradable suture anchors. The type of implant material used (metal vs. biodegradable) did not influence the outcome of arthroscopic rotator cuff repair.

## Data Availability

The dataset supporting the conclusions of this article will be provided upon reasonable request from the corresponding authors.

## Abbreviation

MRI: Magnetic resonance imaging
OSS: Oxford shoulder score
RC: Rotator cuff
RCR: Rotator cuff repair
RCR: Rotator cuff repair
ROM: Range of motion
UCLA: University of California, Los Angeles

## References

[1] Longo UG, Salvatore G, Rizzello G, Berton A, Ciuffreda M, Candela V, Denaro V (2017). The burden of rotator cuff surgery in Italy: a nationwide registry study. Arch Orthop Trauma Surg.

[2] Longo UG, Berton A, Papapietro N, Maffulli N, Denaro V (2012). Epidemiology, genetics and biological factors of rotator cuff tears. Med Sport Sci.

[3] Audigé L, Flury M, Müller AM, Durchholz H, Panel ACC (2016). Complications associated with arthroscopic rotator cuff tear repair: definition of a core event set by Delphi consensus process. J Shoulder Elb Surg.

[4] Longo UG, Franceschi F, Ruzzini L, Spiezia F, Maffulli N, Denaro V (2009). Higher fasting plasma glucose levels within the normoglycaemic range and rotator cuff tears. Br J Sports Med.

[5] Longo UG, Franceschi F, Spiezia F, Marinozzi A, Maffulli N, Denaro V (2011). The low-profile Roman bridge technique for knotless double-row repair of the rotator cuff. Arch Orthop Trauma Surg.

[6] Franceschi F, Longo UG, Ruzzini L, Rizzello G, Maffulli N, Denaro V (2008). Soft tissue tenodesis of the long head of the biceps tendon associated to the Roman bridge repair. BMC Musculoskelet Disord.

[7] Franceschi F, Longo UG, Ruzzini L, Rizzello G, Maffulli N, Denaro V (2007). The Roman bridge: a “double pulley - suture bridges” technique for rotator cuff repair. BMC Musculoskelet Disord.

[8] Longo UG, Lamberti A, Rizzello G, Maffulli N, Denaro V (2012). Synthetic augmentation in massive rotator cuff tears. Med Sport Sci.

[9] Longo UG, Berton A, Khan WS, Maffulli N, Denaro V (2011). Histopathology of rotator cuff tears. Sports Med Arthrosc.

[10] Del Buono A, Oliva F, Longo UG, Rodeo SA, Orchard J, Denaro V, Maffulli N (2012). Metalloproteases and rotator cuff disease. J Shoulder Elb Surg.

[11] Tan CK, Guisasola I, Machani B, Kemp G, Sinopidis C, Brownson P, Frostick S (2006). Arthroscopic stabilization of the shoulder: a prospective randomized study of absorbable versus nonabsorbable suture anchors. Arthroscopy.

[12] Jeong JH, Shin SJ (2009). Arthroscopic removal of proud metallic suture anchors after Bankart repair. Arch Orthop Trauma Surg.

[13] Silver MD, Daigneault JP (2000). Symptomatic interarticular migration of glenoid suture anchors. Arthroscopy.

[14] Kaar TK, Schenck RC, Wirth MA, Rockwood CA (2001). Complications of metallic suture anchors in shoulder surgery: a report of 8 cases. Arthroscopy.

[15] Glueck D, Wilson TC, Johnson DL (2005). Extensive osteolysis after rotator cuff repair with a bioabsorbable suture anchor: a case report. Am J Sports Med.

[16] Kelly JD (2005). Disintegration of an absorbable rotator cuff anchor six weeks after implantation. Arthroscopy.

[17] McBirnie JM, Miniaci A, Miniaci SL (2005). Arthroscopic repair of full-thickness rotator cuff tears using bioabsorbable tacks. Arthroscopy.

[18] Cummins CA, Strickland S, Appleyard RC, Szomor ZL, Marshall J, Murrell GA (2003). Rotator cuff repair with bioabsorbable screws: an in vivo and ex vivo investigation. Arthroscopy.

[19] Ambrose CG, Clanton TO (2004). Bioabsorbable implants: review of clinical experience in orthopedic surgery. Ann Biomed Eng.

[20] Barber FA, Herbert MA, Coons DA, Boothby MH (2006). Sutures and suture anchors--update 2006. Arthroscopy.

[21] Magnusson L, Ejerhed L, Rostgård-Christensen L, Sernert N, Eriksson R, Karlsson J, Kartus JT (2006). A prospective, randomized, clinical and radiographic study after arthroscopic Bankart reconstruction using 2 different types of absorbable tacks. Arthroscopy.

[22] Ejerhed L, Kartus J, Funck E, Köhler K, Sernert N, Karlsson J (2000). A clinical and radiographic comparison of absorbable and non-absorbable suture anchors in open shoulder stabilisation. Knee Surg Sports Traumatol Arthrosc.

[23] Elmlund AO, Kartus J, Rostgård-Christensen L, Sernert N, Magnusson L, Ejerhed L (2009). A 7-year prospective, randomized, clinical, and radiographic study after arthroscopic Bankart reconstruction using 2 different types of absorbable tack. Am J Sports Med.

[24] Milano G, Grasso A, Salvatore M, Saccomanno MF, Deriu L, Fabbriciani C (2010). Arthroscopic rotator cuff repair with metal and biodegradable suture anchors: a prospective randomized study. Arthroscopy.

[25] Ellman H, Hanker G, Bayer M (1986). Repair of the rotator cuff. End-result study of factors influencing reconstruction. J Bone Joint Surg Am.

[26] Murena L, Vulcano E, D'Angelo F, Monti M, Cherubino P (2010). Italian cross-cultural adaptation and validation of the Oxford shoulder score. J Shoulder Elb Surg.

[27] Younis F, Sultan J, Dix S, Hughes PJ (2011). The range of the Oxford shoulder score in the asymptomatic population: a marker for post-operative improvement. Ann R Coll Surg Engl.

[28] Longo UG, Saris D, Poolman RW, Berton A, Denaro V (2012). Instruments to assess patients with rotator cuff pathology: a systematic review of measurement properties. Knee Surg Sports Traumatol Arthrosc.

[29] Haque A, Pal Singh H (2018). Does structural integrity following rotator cuff repair affect functional outcomes and pain scores? A meta-analysis. Should Elb.

[30] Magee T, Shapiro M, Hewell G, Williams D (2003). Complications of rotator cuff surgery in which bioabsorbable anchors are used. AJR Am J Roentgenol.

[31] Kilicoglu O, Demirhan M, Akman S, Atalar AC, Ozsoy S, Ince U (2003). Failure strength of bioabsorbable interference screws: effects of in vivo degradation for 12 weeks. Knee Surg Sports Traumatol Arthrosc.

[32] Pietschmann MF, Fröhlich V, Ficklscherer A, Gülecyüz MF, Wegener B, Jansson V, Müller PE (2009). Suture anchor fixation strength in osteopenic versus non-osteopenic bone for rotator cuff repair. Arch Orthop Trauma Surg.

[33] AmericanAccademyof OrthopaedicSurgeons (1965). Joint motion: method of measuring and recording.

[34] Maffulli N, Longo UG, Franceschi F, Rabitti C, Denaro V (2008). Movin and Bonar scores assess the same characteristics of tendon histology. Clin Orthop Relat Res.

[35] Longo U G, Franceschi F, Ruzzini L, Rabitti C, Morini S, Maffulli N, Denaro V (2007). Characteristics at haematoxylin and eosin staining of ruptures of the long head of the biceps tendon. British Journal of Sports Medicine.

[36] Longo UG, Franceschi F, Ruzzini L, Rabitti C, Morini S, Maffulli N, Denaro V (2008). Histopathology of the supraspinatus tendon in rotator cuff tears. Am J Sports Med.

[37] Longo UG, Franceschi F, Ruzzini L, Rabitti C, Morini S, Maffulli N, Forriol F, Denaro V (2007). Light microscopic histology of supraspinatus tendon ruptures. Knee Surg Sports Traumatol Arthrosc.

[38] Bostman OM (1991). Osteolytic changes accompanying degradation of absorbable fracture fixation implants. J Bone Joint Surg (Br).

[39] Barber FA, Coons DA, Ruiz-Suarez M (2007). Cyclic load testing of biodegradable suture anchors containing 2 high-strength sutures. Arthroscopy.

